# Subjective estimation of cognitive function in mild cognitive impairment: relationship with neurodegenerative and non-degenerative factors

**DOI:** 10.1017/S0033291725102997

**Published:** 2026-01-14

**Authors:** Calum A. Hamilton, Peter Gallagher, Paul C. Donaghy, Joanna Ciafone, Michael Firbank, Gemma Greenfinch, Amanda Heslegrave, Henrik Zetterberg, John-Paul Taylor, Louise M. Allan, John T. O’Brien, Alan J. Thomas

**Affiliations:** 1 Translational and Clinical Research Institute, Newcastle University, Newcastle upon Tyne, UK; 2 University College London Hospitals, UK; 3 University College London, UK; 4 UK Dementia Research Institute, UK; 5 Department of Psychiatry and Neurochemistry, Institute of Neuroscience and Physiology, The Sahlgrenska Academy, University of Gothenburg, Sweden; 6 Clinical Neurochemistry Laboratory, Sahlgrenska University Hospital, Sweden; 7 Hong Kong Center for Neurodegenerative Diseases, Hong Kong; 8 Centre for Brain Research, Indian Institute of Science, India; 9 Wisconsin Alzheimer’s Disease Research Center, University of Wisconsin-Madison, USA; 10 University of Exeter, UK; 11 University of Cambridge, UK

**Keywords:** anosognosia, insight, metacognition, mild cognitive impairment, subjective cognitive complaints

## Abstract

**Background:**

Subjective cognitive complaints are poor predictors of neurodegenerative disease and future dementia. Errors in metacognition, positive or negative differences between actual and perceived performance, may partially explain this. We aimed to assess whether hypothesized indicators of underlying neurodegenerative factors (e.g. hippocampal atrophy) in mild cognitive impairment (MCI) were associated with overestimation of actual cognitive performance, and hypothesized non-degenerative factors (e.g. depression) were associated with underestimation of performance.

**Methods:**

Metacognitive error was estimated from paired subjective and objective cognitive assessments using the Multifactorial Memory Questionnaire and Addenbrooke’s Cognitive Examination – Revised, respectively. A normative model was developed with cognitively healthy older adults (*n* = 36), and applied to individuals with suspected MCI due to Alzheimer’s disease (AD) or MCI with Lewy bodies (total *n* = 88). Theorized predictors of subjective overestimation or underestimation of performance (metacognitive error) were assessed, including demographics, AD biomarkers, and mental and physical ill health. Metacognitive error was also assessed as a predictor of conversion to dementia.

**Results:**

Underestimation of cognitive function was associated with depressive symptoms, anxiety, and self-reported autonomic symptoms. Overestimation of cognitive function was associated with age, hippocampal atrophy, plasma glial fibrillary acidic protein, and subsequent dementia conversion.

**Conclusions:**

Underestimation of cognitive function may reflect functional cognitive changes linked to mental and physical ill health, while overestimation of function may be a marker of neurodegenerative changes. Quantifying metacognitive error may provide a noninvasive screening tool for progressive MCI, requiring investigation in an independent sample.

## Introduction

### Subjective cognitive impairment

Subjective cognitive symptoms – self reported cognitive changes, errors, or difficulty – have been positioned as an early manifestation of developing neurodegenerative diseases, theorized in some situations to precede measurable objective changes in cognitive function, and supported by meta-analyses identifying increased risks of subsequent mild cognitive impairment (MCI) and dementia in people with subjective cognitive impairment (SCI; Mitchell et al., 2014).

However, by far the most common associating factor with SCI is depression, with related psychological and physical factors including anxiety, psychological stress, personality traits such as neuroticism, family isolation, fatigue, and the presence of medical conditions, including diabetes, having also been identified as possibly negatively affecting cognitive self-perception in healthy older adults (Brett et al., 2023; Roh, Dan, & Kim, 2021; Smit et al., 2024).

### Mismatch between subjective and objective performance

Consequently, subjective reports of cognitive symptoms may not correlate with objective measures, with people often under-estimating their own abilities. Such alterations of global metacognition (self-perception of cognitive ability) have been identified as possible mechanisms in functional cognitive disorders (FCDs; Bhome et al., 2022), which are a common cause of subjective cognitive complaints (Bharambe & Larner, 2018; Pennington, Hayre, Newson, & Coulthard, 2015).

Conversely, anosognosia – absence of insight into one’s own symptoms – is a common feature of many neurological and psychiatric conditions, including Alzheimer’s disease (AD) and related disorders at both prodromal and clinically manifest stages (Mak et al., 2015; Sunderaraman & Cosentino, 2017). Its presence may therefore be a clinical marker of underlying neurodegenerative pathological changes, and consequently indicative of a worse prognosis (Wilson et al., 2015).

### Metacognitive error

Both cognitive anosognosia and SCI/FCD may be conceptualized as opposing ends on a single continuum of global metacognitive error. Positive metacognitive error represents a subjective overestimation of one’s own objective cognitive abilities, whereas negative error represents a subjective underestimation of these. Positive or negative metacognitive error may inform us about the underlying contributions to an observed cognitive impairment, with positive error being a marker of underlying neurodegenerative pathology and consequently worse prognosis, whereas negative error may be a marker of functional cognitive changes and FCD (Cabreira et al., 2025).

Metacognitive error may be estimated and quantified by pairing subjective and objective cognitive assessment. Previous research has demonstrated the value of using paired subjective-objective cognitive assessment to estimate local metacognitive processes using a signal detection theory framework (Seow, Rouault, Gillan, & Fleming, 2021),

Global metacognitive processes may be quantified using simple pen-and-paper assessments, as previously demonstrated in MCI and other cognitive disorders (Bhome et al., 2022; Lenehan, Klekociuk, & Summers, 2012). However, this method does not inherently account for objective performance: subjective cognitive complaints may be incongruent for an individual with objectively good cognitive performance, but congruent for another individual with worse objective performance. Regression-based approaches may enable quantification of global metacognitive error with such simple tasks (Miskowiak et al., 2016; Wilson et al., 2015).

### Aims and hypotheses

We therefore aimed to build on this previous work by estimating global metacognitive error through calculating the disparity between self-rated and objective global cognitive performance, and by testing whether this estimated metacognitive error was associated with key psychological, biological, and health-related factors.

We hypothesized that global metacognitive error would be positively associated with markers of neurodegenerative disease (hippocampal atrophy and blood-based biomarkers of AD and neurodegeneration) and negatively associated with concurrent psychological symptoms (anxiety and depressive symptoms) as well as physical health symptoms.

## Methods

### Design

We undertook a secondary analysis of cross-sectional baseline data from a longitudinal study of MCI. Data were drawn from the ‘SUPErB’ cohort study, which recruited individuals with suspected MCI-LB or MCI-AD, as well as healthy older adults, from North East England (Donaghy et al., 2022). Primary aims of this study were to examine dementia with Lewy bodies (DLB) indicative biomarkers in differentiating MCI-LB from MCI-AD, as well as the prospective evolution of these conditions. In this secondary analysis, we accessed data from baseline and follow-up assessments to examine the disparity between objective and subjective cognitive assessment at baseline in a heterogeneous MCI sample.

### Participants

Individuals aged 60 years or older with a diagnosis of MCI were recruited from older person’s healthcare services in North East England, from 2016 to 2019.

After providing written, informed consent, participants underwent detailed screening for inclusion in the study, including objective cognitive and neuropsychiatric assessment, clinical interview, and assessment of independent function supported by an informant, where available. A three-person panel made the final clinical diagnosis using all available information to confirm the diagnosis of MCI, as well as determining the consensus MCI subtype (Albert et al., 2011; McKeith et al., 2020). Exclusion criteria included the presence of dementia, preexisting Parkinson’s disease for >12 months prior to onset of cognitive symptoms, any suspected MCI etiology other than MCI due to AD (MCI-AD) or MCI with Lewy bodies (MCI-LB), being medically unstable due to undergoing investigation or treatment for any major medical comorbidity, or experiencing an active episode of major psychiatric illness.

Healthy older adults were also recruited from friends and family of MCI participants, and from targeted advertisements through local research networks. They were required to be aged 60 years or older, and free from any known neurological condition.

Written, informed consent was obtained from all participants.

### Diagnostic investigation and imaging

Participants underwent detailed medical review during screening, providing clinical research notes on the presence of cognitive symptoms, severity of any functional impairment, and presence of additional clinical features, including core clinical features and supportive clinical features of DLB.

Two indicative biomarker investigations for DLB were administered to all participants: FP-CIT SPECT imaging of dopamine transporter uptake to the striatum, and metaiodobenzylguanidine (MIBG) cardiac scintigraphy, as detailed previously (Roberts et al., 2021a; Roberts et al., 2021b). Dopamine transporter imaging was undertaken using a Siemens gamma camera with low energy collimator, between 3 h and 6 h after intravenous injection of 123I-FP-CIT (GE Healthcare, UK). FP-CIT images were visually rated by an experienced panel of imaging analysts as normal or abnormal, blind to clinical diagnosis. Cardiac images were captured by a 10-min scan between 3.5 h and 4.5 h after intravenous injection of 123I-MIBG, using a Siemens gamma camera with medium energy collimators. Heart-to-mediastinum ratio was quantified from MIBG images, with a cut-off of 1.85 used to differentiate normal from abnormal uptake (based on local normative data). These biomarker investigation findings were incorporated into differential MCI classifications (see below).

Participants also underwent structural MRI at baseline, as previously described (Firbank et al., 2021), providing quantified measurement of hippocampal volume and total intracranial volume. To estimate hippocampal atrophy, raw hippocampal volume measures were adjusted for total intracranial volume using a control group residual-based approach (O’Brien et al., 2011), regressing hippocampal volume on total intracranial volume to account for expected proportionality with a freely estimated offset. Resulting values were standardized and inverted to represent atrophy as the difference between expected and actual hippocampal volume, given each individual’s total intracranial volume. MR images were visually inspected for evidence of cerebrovascular disease as a cause for exclusion, but not otherwise incorporated into etiology differential classifications.

### Diagnoses

After the baseline study medical review, participants’ research notes were reviewed independently by a three-person panel of experienced old age psychiatrists (PCD, JPT, and AJT). The panel ratified the health service diagnosis of MCI, and any participants whose health service MCI diagnosis was not ratified by the panel were excluded.

The panel then provided each MCI patient with a subtype classification as either MCI-AD or MCI-LB based on consensus clinical or research criteria (respectively; Albert et al., 2011; McKeith et al., 2020). As previously described, MCI classifications incorporated the presence or absence of core clinical features of LB disease alongside indicative biomarkers to identify probable MCI-LB (≥2 core clinical features, or 1 core clinical feature with ≥1 abnormal indicative biomarker), possible MCI-LB (1 core clinical feature with no abnormal indicative biomarker, or 0 core clinical features with ≥1 abnormal indicative biomarker) or MCI-AD (no core clinical features or indicative biomarkers of DLB, and ‘evidence of decline consistent with AD with no evidence for another etiology’; Albert et al., 2011; Donaghy et al., 2018; Donaghy et al., 2022).

Individuals with a suspected non-AD or non-LB etiology were excluded at baseline. Additional cases for exclusion were identified occasionally at follow-up, including progressive supranuclear palsy, vascular cognitive impairment, and subjective-only cognitive impairment (Hamilton et al., 2024a). Any such cases were included in this baseline analysis to avoid introducing circularity from future observations.

In the absence of any prior reason to suspect there would be different metacognitive profiles in MCI-AD and MCI-LB, MCI was investigated as a single combined group in the primary analysis. Subgroup differences were explored in secondary analyses.

### Cognitive assessment

Within the wider detailed study assessment as reported previously, two measures were administered to assess objective and subjective global cognitive performance, respectively: the Addenbrooke’s Cognitive Examination – Revised (ACE-R), a 100-point global cognitive screening test, and the Multifactorial Memory Questionnaire (MMQ; Troyer & Rich, 2002), a questionnaire assessing global metacognition and subjective cognitive symptoms. In both assessments, higher values reflect better objective or perceived cognitive performance.

The MMQ contains three subscales, reflecting satisfaction, self-rated ability, and use of mnemonic strategies. As previously described by Bhome et al. (2022), while the former two are likely to reflect global metacognitive processes and are highly correlated, strategy use has a less clear relationship with cognitive dysfunction and was excluded from this analysis.

To estimate global metacognitive error, a normative model was first constructed incorporating baseline MMQ and ACE-R scores from healthy controls. This normative model was then used to estimate expected MMQ scores from ACE-R values for each MCI patient, with residuals reflecting the (standardized) difference between the predicted and actual MMQ value (see Supplementary Figure S1).

Positive metacognitive error values therefore reflect a relative overconfidence in own cognitive ability, and negative metacognitive error values reflect a relative underconfidence.

### Predictors of metacognitive error

Measures of several hypothesized predictors of global metacognitive error were available at study baseline:

Depressive symptoms were measured with the 15-item Geriatric Depression Scale (GDS-15; D’Ath et al., 1994), administered to the patient.

Anxiety symptoms were measured with the informant-rated neuropsychiatric inventory (NPI) anxiety subscale (Cummings et al., 1994).

Overall physical health was measured using the Cumulative Illness Rating Scale-Geriatric (CIRS-G; Miller & Towers, 1991), completed by a medically qualified researcher, and perceived autonomic symptom burden was quantified using the Composite Autonomic Symptom Score-31 (COMPASS-31) scale (Sletten et al., 2012), administered to the patient.

Also incorporated were estimated hippocampal atrophy (see above) and concentrations of several plasma biomarkers of AD (Aβ42/40 ratio and pTau-181), neurodegeneration (neurofilament light [NfL] chain) and astrocyte activity (glial fibrillary acidic protein [GFAP]), measured using ultrasensitive single-molecule array technology (Quanterix, Billerica, MA, USA), as previously described (Hamilton et al., 2023).

Associations of demographic variables age, education, and socioeconomic status (English Indices of Multiple Deprivation), and overall global cognitive function, were also examined.

### Analyses

Associations between continuous metacognitive error values and hypothesized predictors were assessed with univariable and multivariable standardized linear models with additional adjustment for age. Predictors anticipated to be highly skewed were log-transformed to account for their multiplicative nature.

All MCI cases or healthy controls who completed the MMQ assessment at baseline were included for analysis. Those with wholly missing MMQ were excluded.

All statistical analyses were undertaken using R (Version 4.5.0).

### Ethics

The authors assert that all procedures contributing to this work comply with the ethical standards of the relevant national and institutional committees on human experimentation and with the Helsinki Declaration of 1975, as revised in 2013. All procedures involving human subjects/patients were approved by the National Research Ethics Service Committee North East – Newcastle & North Tyneside 2 (No. 15/NE/0420).

## Results

Retrospectively, the MCI group consisted of MCI-AD (*n* = 29), possible MCI-LB (*n* = 14), probable MCI-LB (*n* = 38), progressive supranuclear palsy (*n* = 1), vascular cognitive impairment (*n* = 2), and subjective-only cognitive impairment (SCI; *n* = 4). MCI cases who were reclassified as SCI showed a sustained improvement in objective function that was not consistent with a neurodegenerative process, and was not explained by a positive response to initiating antidementia medication, but they continued to report persistent subjective cognitive symptoms. Subgroup analyses did not identify any significant differences between MCI-LB and MCI-AD groups for MMQ Satisfaction (*p* = 0.708), Ability (*p* = 0.314), or Strategy (*p* = 0.567) scores. Overall, people with MCI typically reported lower memory satisfaction (*p* < 0.001) and ability (*p* < 0.001) than healthy controls (*n* = 36), and higher use of mnemonic strategies (*p* = 0.009; see Table 1).

**Table 1.** Baseline characteristics of sample

Characteristic	MCI *N* = 88^1^	Control *N* = 36^1^
Age (years)	75 (7)	75 (7)
Female gender	29 (33%)	9 (25%)
ACE-R total score/100	82 (10)	93 (4)
MMQ satisfaction score/72	31 (14)	53 (10)
MMQ ability score/80	37 (13)	57 (9)
MMQ strategy score/76	32 (11)	26 (12)
Missing	3	0

1 Mean (SD); n (%).

### Normative models

**Figure 1.**
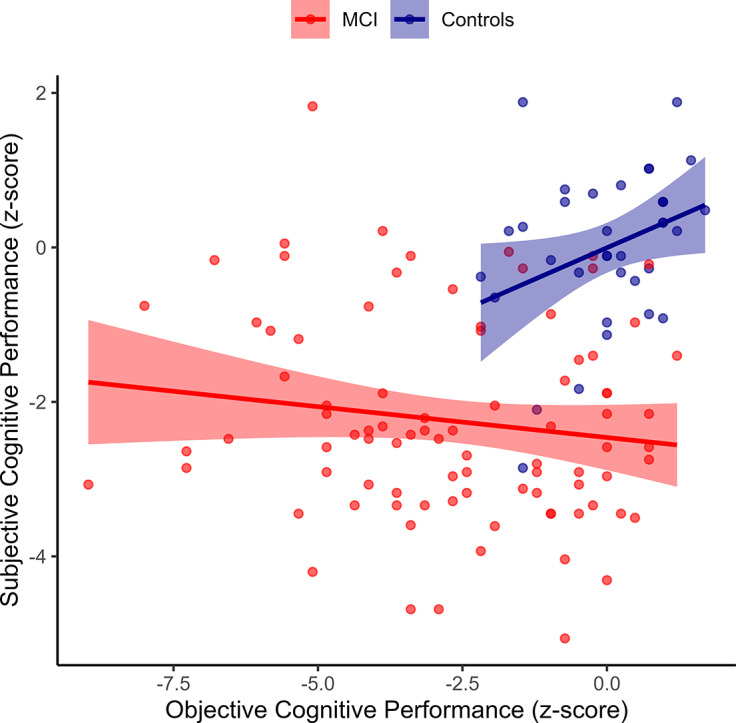
Associations between objective and subjective cognitive performance in healthy older adults versus MCI.

MMQ Ability + Satisfaction total score was positively associated with total score on the ACE-R for healthy older adults (*r* = 0.33, *p* = 0.043), but not MCI patients (*r* = −0.14, *p* = 0.189; see Figure 1).

Shapiro–Wilk tests indicated that residuals from the normative model were not significantly non-Gaussian (*p* = 0.336), metacognitive error derived from the normative model was also not significantly non-Gaussian for MCI patients (*p* = 0.220), and there was no significant difference between MCI-AD and MCI-LB subgroups (*p* = 0.680).

For the structural MRI normative model, hippocampal volume was positively associated with total intracranial volume in healthy controls (*r* = 0.52, *p* = 0.001) and MCI patients (*r* = 0.40, *p* < 0.001), with approximately normally distributed residuals in controls (*p* = 0.349) and MCI patients (*p* = 0.236).

### Associations between clinical features and metacognitive error

**Figure 2.**
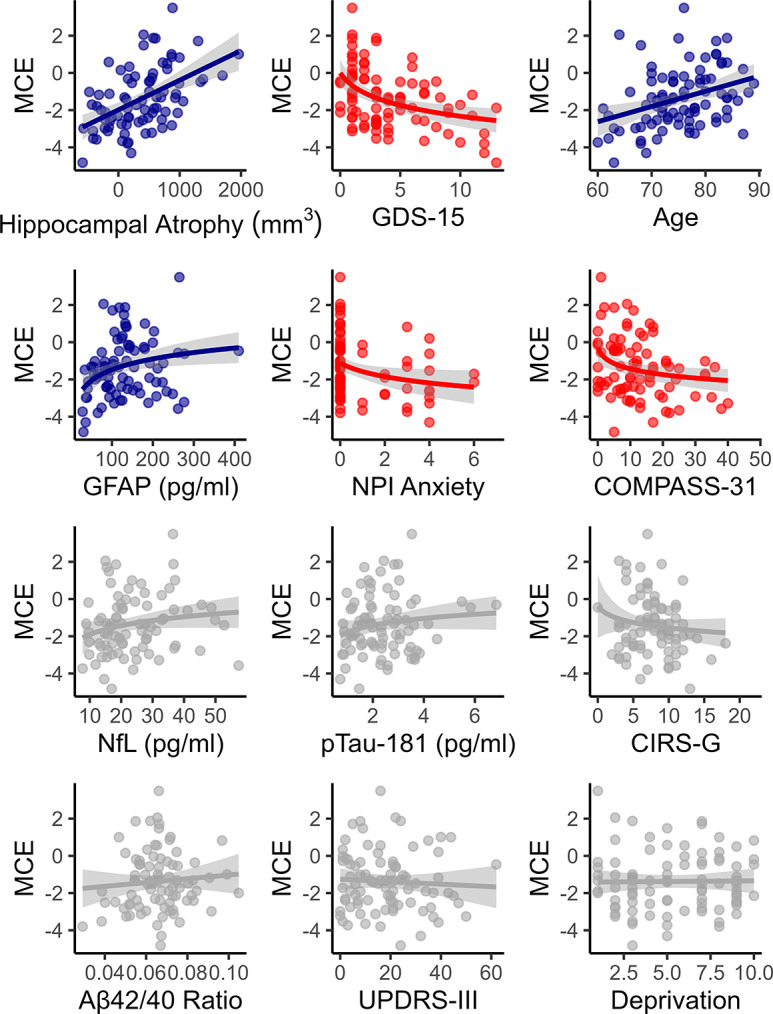
Associations between metacognitive error (MCE) and theorized predictors. Red points denote significant predictors of negative MCE, blue points denote significant predictors of positive MCE, and gray points denote nonsignificant predictors.

In unadjusted analyses (see Table 2 and Figure 2), estimated metacognitive error was positively associated with age, hippocampal atrophy, and plasma GFAP concentrations, and negatively associated with GDS-15 depression scores, NPI anxiety subscale scores, and COMPASS-31 autonomic symptom scores.

**Table 2.** Unadjusted and mutually adjusted associations between metacognitive error and hypothesized predictor variables

	Univariable associations		Multivariable associations	
	Unadjusted β (95% CI)	*P*-value	Adjusted β (95% CI)	*P*-value
*Demographic*				
Age	0.36 [0.15–0.57]	<0.001	0.19 [−0.12 to 0.49]	0.228
Education	−0.09 [−0.33 to 0.14]	0.439	−0.17 [−0.43 to 0.08]	0.180
Socioeconomic deprivation	0.01 [−0.21 to 0.23]	0.911	−0.28 [−0.51 to −0.05]	0.016
*Biomarkers*				
Hippocampal atrophy	0.52 [0.32–0.72]	<0.001	0.43 [0.19–0.67]	<0.001
Plasma Aβ42/40 ratio	0.08 [−0.13 to 0.29]	0.441	0.13 [−0.07 to 0.33]	0.197
Plasma pTau–181 concentration*	0.16 [−0.05 to 0.37]	0.137	0.12 [−0.17 to 0.40]	0.417
Plasma NfL concentration*	0.19 [−0.03 to 0.41]	0.087	0.01 [−0.32 to 0.33]	0.978
Plasma GFAP concentration*	0.31 [0.09–0.52]	0.005	0.07 [−0.24 to 0.39]	0.948
*Mental health*				
Geriatric Depression Scale – 15*	−0.40 [−0.59 to −0.21]	<0.001	−0.34 [−0.59 to −0.09]	0.010
NPI Anxiety Subscale*	−0.28 [−0.51 to −0.05]	0.016	−0.12 [−0.34 to 0.09]	0.266
*Physical health*				
CIRS-G Total Score*	−0.16 [−0.42 to 0.1]	0.237	−0.14 [−0.39 to 0.12]	0.294
Compass–31 Score*	−0.30 [−0.52 to −0.08]	0.009	−0.02 [−0.28 to 0.24]	0.879
UPDRS-III Motor Score*	−0.06 [−0.27 to 0.16]	0.595	−0.10 [−0.35 to 0.15]	0.417

* Log transformed.

Mutually adjusted analyses indicated that the strongest unique associations were a positive association between metacognitive error and hippocampal atrophy (β = 0.34, *p* = 0.004) and negative associations with both GDS-15 (β = −0.34, *p* = 0.002) and socioeconomic disadvantage (β = −0.28, *p* = 0.016).

### MCI prognosis and diagnostic revision

Metacognitive error was positively associated with progression to dementia within 3 years of baseline in a univariable model (OR = 1.38 per 1 *z*-score, *p* = 0.038); however, including this variable in a multivariable prediction model did not improve over a null model incorporating global cognitive function only (*p* = 0.348).

Four individuals were subsequently reclassified as SCI at follow-up (blind to MMQ scores). Three of these four had pronounced negative metacognitive error at greater than 2 *z*-scores (*z* = −3.7, −3.0 and −2.4) at baseline, although the fourth accurately appraised their own cognitive performance (*z* = −0.2).

### Sensitivity analyses

Several sensitivity analyses were conducted to examine possible subgroup differences. Excluding those who were not considered, after follow-up, to be either MCI-AD or probable MCI-LB did not meaningfully change the results: there were significant positive associations between metacognitive error and hippocampal volume (β = 0.48, *p* < 0.001), age (β = 0.34, *p* = 0.005), and plasma GFAP (β = 0.25, *p* = 0.045), and significant negative associations with GDS-15 (β = −0.35, *p* = 0.003), NPI anxiety (β = −0.32, *p* = 0.015), and COMPASS-31 (β = −0.27, *p* = 0.029). Metacognitive error was not significantly predictive of dementia conversion after follow-up SCI cases were removed (OR = 1.34, *p* = 0.059), suggesting that these cases at least partially contributed to this effect, although the direction and size of the effect were largely comparable.

Subgroup analyses in MCI-AD and MCI-LB provided divergent results for some predictors. In MCI-AD, metacognitive error was strongly and positively related to hippocampal atrophy (β = 0.59, *p* < 0.001), and negatively associated with socioeconomic disadvantage (β = −0.40, *p* = 0.031). In MCI-LB, metacognitive error was moderately and positively related to hippocampal atrophy (β = 0.44, *p* = 0.008), and negatively associated with GDS-15 (β = −0.42, *p* = 0.008) and NPI anxiety (β = −0.37, *p* = 0.031).

Several significant predictors in the overall MCI group showed similar but nonsignificant or variably significant effects in both individual groups, suggesting that these were consistent but limited by smaller sample sizes of the subgroup analysis (specifically, depression, anxiety, age, autonomic symptoms, and plasma GFAP; see Supplementary Figure S2).

Additional sensitivity analyses indicated that metacognitive error was not significantly associated with gender (*p* = 0.844).

We assessed possible mediating effects of relevant medications. Use of antidepressants (β = −0.35 [−1.1 to 0.36], *p* = 0.326), anxiolytics (β = 0.06 [−2.2 to 2.3], *p* = 0.955), or cholinesterase inhibitors/memantine (β = 0.19 [−0.45 to 0.82], *p* = 0.563) was not significantly associated with metacognitive error when adjusting for depressive, anxious, or cognitive symptom severity, respectively.

## Discussion

In this study, we aimed to assess whether global metacognitive error in people with MCI, estimated by differences between coincident subjective- and objective cognitive assessment in individuals, was associated with several factors theorized to reflect structural and functional cognitive changes.

We found that MCI patients with more severe hippocampal atrophy and higher plasma GFAP concentrations typically overestimated their cognitive function (relative to the normal model), whereas those with higher scores on symptoms of depression, anxiety, and autonomic dysfunction typically underestimated their cognitive function. Those who overestimated their cognitive abilities were more likely to have developed dementia after 3 years of follow-up.

These findings suggest that overall, self-rated cognitive performance in MCI is largely unrelated to the severity of objective performance deficits. This has previously been observed in MCI (Chung & Man, 2009; Lenehan et al., 2012), as well as a variety of non-neurodegenerative clinical conditions, including people with major depressive disorder (Serra-Blasco et al., 2019), survivors of intensive care units (Brück et al., 2019), and community-dwelling older adults (Topiwala et al., 2021), suggesting that this is not an issue specific to neurodegenerative conditions.

We further show that discrepancy between subjective and objective global cognitive performance may be informative as to underlying neurodegenerative- and non-degenerative processes in MCI.

Subjective overestimation of objective cognitive performance (positive metacognitive error) may be a marker of neurodegenerative disease and consequently dementia risk. This is consistent with the observed association between anosognosia and metacognitive alterations with neurodegenerative and cerebrovascular pathology (Hallam et al., 2020; Wilson et al., 2015). Positive metacognitive error may therefore be related to underlying dementia pathology.

In contrast, mental and physical ill health – specifically depression, anxiety, and autonomic symptoms – were associated with a subjective underestimation of objective cognitive performance. This is consistent with previous observations of an association between subjective cognitive complaints and depression (Dong et al., 2024; Godoy-González et al., 2023; Smith et al., 2023; Topiwala et al., 2021; Wei et al., 2019), anxiety (Brett et al., 2023; Godoy-González et al., 2023), and autonomic symptoms (Sander et al., 2017), in several clinical and nonclinical populations. Negative metacognitive error may therefore be related to lowered perceived self-efficacy either as an outcome of mental and physical ill health, or as a manifestation of some other shared underlying cause such as FCD (Bhome et al., 2019), for which internal inconsistency (e.g. between self-reported and objectively tested cognitive performance) is a proposed positive diagnostic feature (Ball et al., 2020).

While we did not have data available from individuals with diagnosed FCD for direct comparison, our data indicated that individuals in this cohort varied in their functional risk factors, neurodegenerative markers, and progression. Some individuals have a highly neurodegenerative presentation (including elevated blood-based biomarkers, hippocampal atrophy, anosognosia, and rapid dementia onset), while others may have a more mixed presentation (including anxiety, depression, worse self-reported physical health, but lower dementia risk).

While this requires direct comparison between neurodegenerative MCI and FCD, this does tentatively validate previous work suggesting that metacognitive errors may be useful as positive markers of functional cognitive changes. Currently, ruling out AD may not necessarily help diagnose FCD, since biomarker (e.g. pTau) negativity could also be consistent with pure DLB or vascular cognitive impairments. Developing and validating a low-cost and noninvasive clinical screening tool to quantify metacognitive error in this manner may complement these emerging biomarkers.

Comparison of two different MCI subtypes, and with the wider literature, suggests that neither positive nor negative metacognitive errors described are specific to any particular condition. This single bidirectional measure may therefore be helpful for screening and quantification of severity of both neurodegenerative and non-degenerative factors in suspected cognitive disorders, regardless of underlying etiology.

There are several limitations to acknowledge and address in future research. The wider literature indicates that physical frailty and multimorbidity are associated with subjective cognitive complaints (Gifford et al., 2019; Jacob, Haro, & Koyanagi, 2019) and may therefore contribute to dementia diagnostic rates through nonpathological pathways (Hamilton et al., 2024b). We found an association between metacognitive error and self-rated autonomic symptoms in this cohort, but not with clinician-rated CIRS-G. It is therefore unclear whether this represents an association that is specific to autonomic symptoms rather than wider multimorbidity, or whether the self-rated nature of the COMPASS-31 is important. SCI may imply a specific diagnosed condition whereas subjective symptoms could be present in any condition. This would be consistent with a shared mechanism underlying both, with abnormal self-monitoring processes manifesting as greater self-reported cognitive and physical symptoms relative to objectively assessed severity of these.

While we found anticipated associations between positive metacognitive error and objective biomarkers of organic brain disease (GFAP and hippocampal atrophy), this was not consistent across all considered markers, with no significant associations with pTau-181 and NfL (although the associations with NfL were comparable to those seen with GFAP in MCI-AD). This warrants further exploration, but may be explained by the specific dynamics of each respective marker, with some becoming abnormal early in MCI, but not necessarily being proportional to severity/stage (e.g. pTau-181: Thomas et al., 2022), others being proportional to severity/stage but still developing in MCI (e.g. NfL: Chatterjee et al., 2023; Hamilton et al., 2023), whereas GFAP and hippocampal volumes may become abnormal both early in MCI, and proportionally to disease severity (Chatterjee et al., 2023; Firbank et al., 2021).

These data were drawn from a larger longitudinal cohort study, but global metacognition was assessed only at study baseline. This precluded us from examining how metacognitive error evolved over the disease course, and whether this was associated cross-sectionally or longitudinally with changes in physical, mental, or cognitive health. Metacognitive judgments may vary over time or with disease progression. Future research may therefore benefit from repeated reassessment of both subjective and objective cognitive performance.

The MMQ is a memory-specific measure (Troyer & Rich, 2002). While MCI-AD and AD are typically characterized by objective memory dysfunction, MCI-LB, DLB, atypical AD, and other non-AD dementias may have a different pattern of cognitive deficits. This may limit the utility of the MMQ in assessing global metacognitive changes for individuals with a broader pattern of cognitive impairments, for example, with relatively worse executive, language, or visuospatial functions. Such individuals could appear to overestimate their global cognitive ability due to mismatch between the queried and affected domains rather than any metacognitive alterations. There may therefore be value in utilizing either a domain-general global metacognition questionnaire or one which addresses multiple specific cognitive domains, to provide clearer insight into the role of metacognition in non-amnestic conditions.

All included patients met criteria for either MCI-AD or MCI-LB at baseline, although a small number were, retrospectively, reclassified after several years of follow-up (Hamilton et al., 2024a). We included these participants here according to their original MCI classification, to avoid introducing any circularity from future observations (e.g. reclassification as SCI at follow-up). Sensitivity analyses indicated that the inclusion or exclusion of these cases did not meaningfully change the findings. Four participants showed objective cognitive improvement, eventually being reclassified as subjective-only cognitive impairment at follow-up; there were insufficient numbers to quantitatively compare this subgroup to others, although three of the four showed pronounced negative metacognitive error at baseline, tentatively validating the possible prognostic utility of this measure.

Building on previous work using the MMQ, this analysis benefited from adjustment of subjective cognitive scores for objective cognitive performance with regression-based norms derived from healthy controls to estimate metacognitive error specifically, rather than subjective cognitive function alone. However, this method must linearly extrapolate beyond the bounds of control data. If there is a nonlinear relationship between objective and subjective cognition function, then this will not be captured by this method. However, estimated metacognitive error was approximately normally distributed, supporting that this measure was fit for purpose in this cohort.

Both MCI subgroups, and the neurodegenerative/non-degenerative indicators included, were participants or markers of convenience, derived from a larger preexisting cohort rather than chosen by design. Future research may benefit from incorporating a larger mixed cohort more representative of the range of individuals seen in memory clinics, and incorporating specific measures to assess both subjective- and objective factors relating to cognition, mental and physical health, and neurodegeneration.

We explored whether use of relevant medications (antidepressants, anxiolytics, cholinesterase inhibitors, or memantine) might be associated with differences in metacognitive function, and did not find evidence to support this. However, this analysis is limited by the nonrandom allocation of medications in this observational study. It may be prudent to assess changes in insight into cognitive performance as a secondary outcome measure in appropriately designed intervention studies to assess this in future research.

## Conclusions

Subjective and objective cognitive assessments may be combined to estimate the degree of discordance between the two. Subjective overestimation of objective cognitive performance in individuals with MCI is associated with markers of neurodegenerative pathology and predictive of disease progression, whereas subjective underestimation of objective performance is associated with mental and physical ill health. Metacognitive errors may therefore provide insights into the underlying factors and possible prognosis of MCI.

## Supporting information

10.1017/S0033291725102997.sm001 Hamilton et al. supplementary material Hamilton et al. supplementary material

## Data Availability

Data from the cohorts used in these analyses are available through the Dementias Platform UK data portal. R code to replicate these analyses is available upon request from the corresponding author.

## Supplementary material

The supplementary material for this article can be found at http://doi.org/10.1017/S0033291725102997.
