# Supplementary material for: Subjective estimation of cognitive function in mild cognitive impairment: relationship with neurodegenerative and non-degenerative factors
Source: Psychol Med. 2026 Jan 14;56:e19. doi: 10.1017/S0033291725102997 (PMC12885339; doi:10.1017/S0033291725102997)
Supplement: Hamilton et al. supplementary material [file S0033291725102997sup001.docx]

**
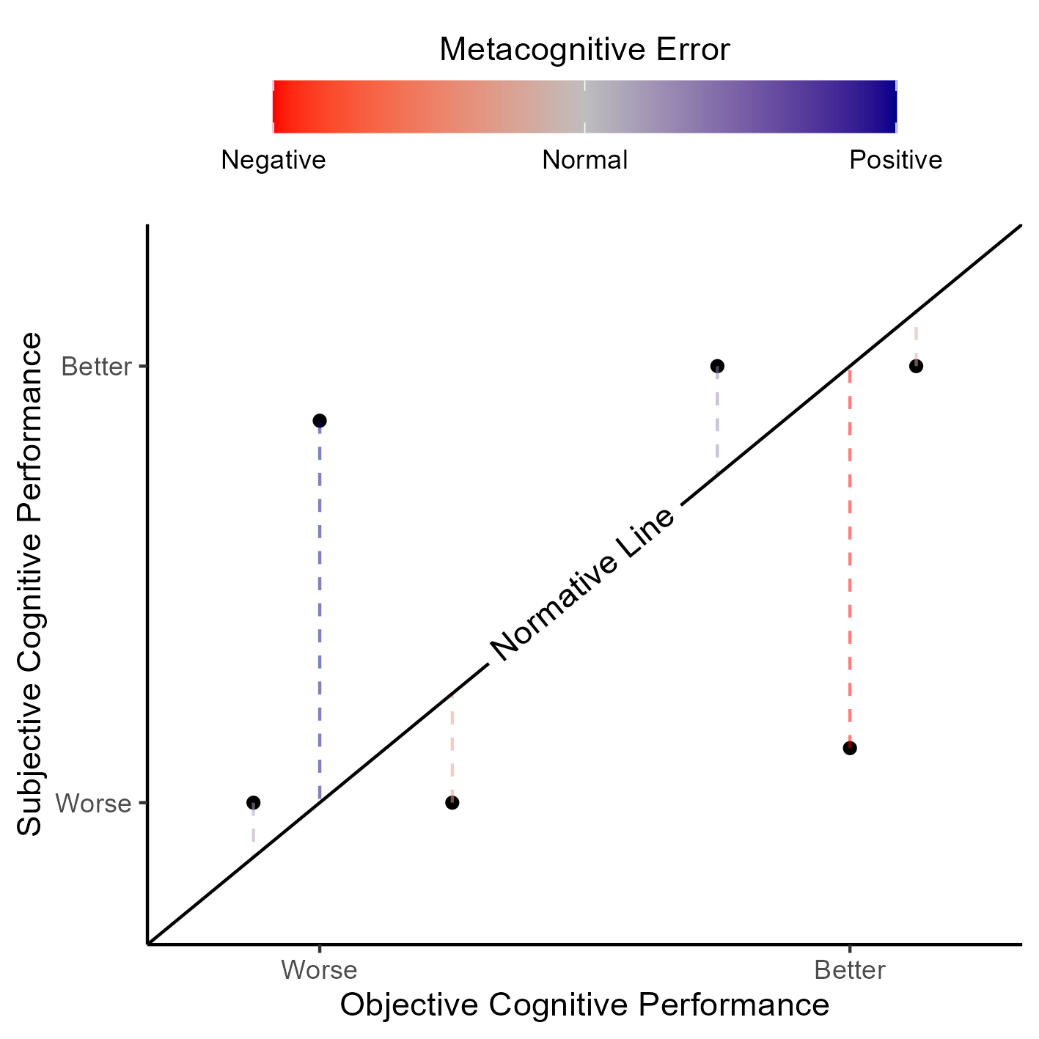
**

**Supplementary Figure S1. Conceptual model for estimating metacognitive error from subjective and objective cognitive performance.**

**
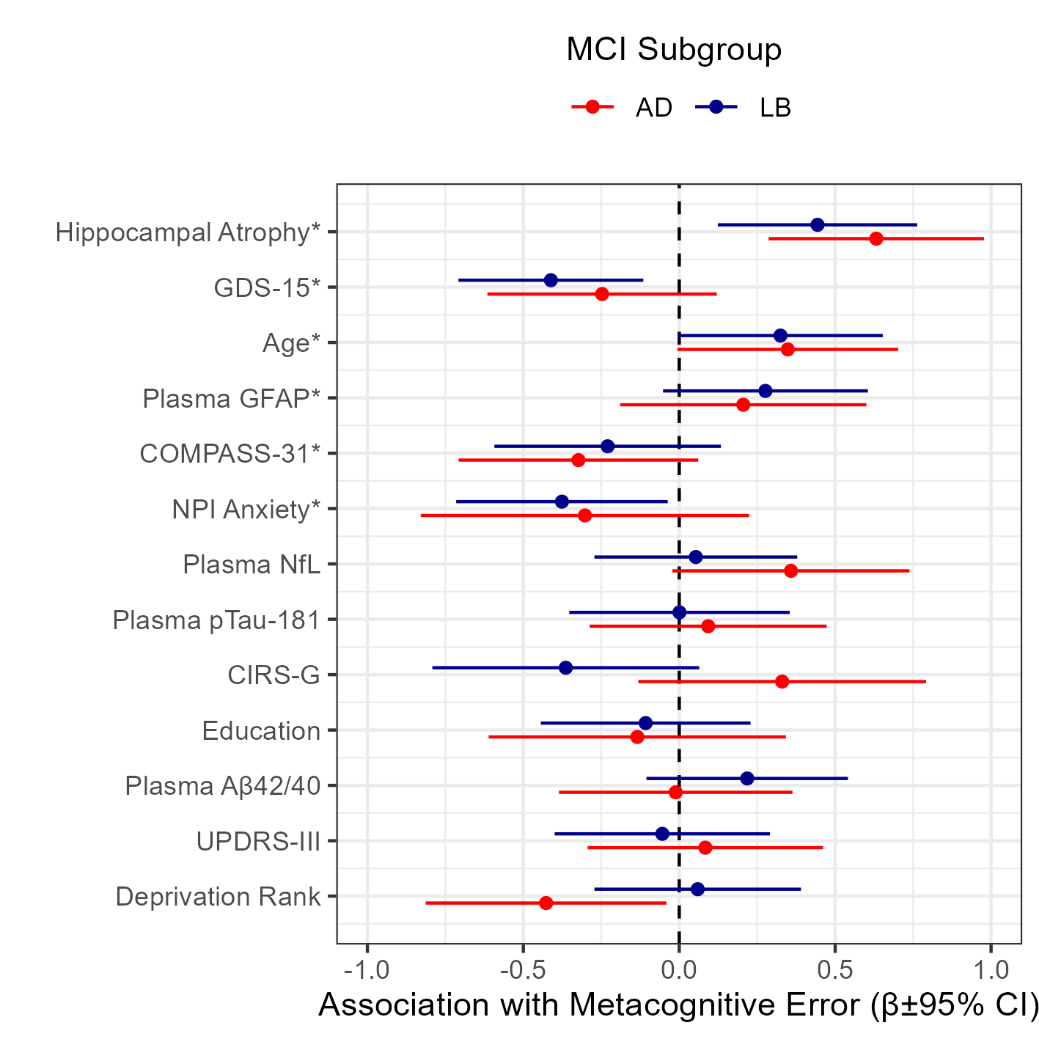
**

**Supplementary Figure S2. Subgroup analysis of factors associated with metacognitive error in MCI, *significant predictors in full group model.**
